# Understanding Driving as an Everyday Activity: International Perspectives

**DOI:** 10.1093/geroni/igaa057.2592

**Published:** 2020-12-16

**Authors:** Anne Dickerson, Isabelle Gelinas, Moon Choi

## Abstract

This international symposium brings together leading occupational therapy researchers from around the world with a shared focus is on evaluating and improving the driving performance of older adults to decrease their crash risk and facilitate their community participation. In this session, five groups of international scholars will share their collective and individual research outcomes for driving as a means of community mobility. The first presentation will outline their collective international, cross-sectional study of 247 older adults from seven countries where the impact of driving on out-of-home mobility was compared. Each presentation that follows will then present results from innovative studies of ways in which to assess and address fitness to drive in older adulthood. Our discussant will summarize the potential expansion of the current work and engage the audience through interactive questions. Transportation and Aging Interest Group Sponsored Symposium.

